# Mesenchymal Stem Cell Therapy Stimulates Endogenous Host Progenitor Cells to Improve Colonic Epithelial Regeneration

**DOI:** 10.1371/journal.pone.0070170

**Published:** 2013-07-29

**Authors:** Alexandra Sémont, Christelle Demarquay, Raphaëlle Bessout, Christelle Durand, Marc Benderitter, Noëlle Mathieu

**Affiliations:** Laboratory of Radiopathology and Experimental Therapeutics, Institute for Radiological Protection and Nuclear Safety, Fontenay-aux-Roses, France; University of Medicine and Dentistry of New Jersey, United States of America

## Abstract

Patients who undergo pelvic radiotherapy may develop severe and chronic complications resulting from gastrointestinal alterations. The lack of curative treatment highlights the importance of novel and effective therapeutic strategies. We thus tested the therapeutic benefit of mesenchymal stem cells (MSC) treatment and proposed molecular mechanisms of action. MSC efficacy was tested in an experimental model of radiation-induced severe colonic ulceration histologically similar to that observed in patients. In this model, MSC from bone marrow were administered intravenously, immediately or three weeks (established lesions) after irradiation. MSC therapy reduces radiation-induced colonic ulceration and increases animal survival. MSC treatment induces therapeutic efficacy whatever the time of cell infusion. Infused-MSC engraft in the colon but also increase endogenous MSC mobilization in blood that have lasting benefits over time. *In vitro* analysis demonstrates that the MSC effect is mediated by paracrine mechanisms through the non-canonical WNT (*Wingless* integration site) pathway. In irradiated rat colons, MSC treatment increases the expression of the non-canonical WNT4 ligand by epithelial cells. The epithelial regenerative process is improved after MSC injection by stimulation of colonic epithelial cells positive for SOX9 (SRY-box containing gene 9) progenitor/stem cell markers. This study demonstrates that MSC treatment induces stimulation of endogenous host progenitor cells to improve the regenerative process and constitutes an initial approach to arguing in favor of the use of MSC to limit/reduce colorectal damage induced by radiation.

## Introduction

Pelvic radiotherapy is an established part of treatment of both primary and recurrent pelvic malignancies, including colorectal, urologic, and gynecologic cancers. The efficacy of radiotherapy requires an optimal compromise between tumor control and toxicity to healthy, non-neoplastic tissues. As a result of pelvic radiotherapy, non-neoplastic tissue present in the irradiation field near the tumor can be damaged, leading to acute and/or chronic symptoms, the condition labeled as “pelvic-radiation disease” by Andreyev et *al.* [1]. Advances in the quality of radiation treatment have improved tumor control, increasing the number of cancer survivors suffering from treatment-related adverse effects. Gastrointestinal symptoms induced by chronic toxicity of irradiation have the greatest effect on patient quality of life. It is estimated that 90% of patients subjected to pelvic radiotherapy develop acute side effects (nausea, alternation of diarrhea and constipation, vomiting and abdominal pain) with permanent changes to their bowel habits. After 20 years, 20% of patients develop severe late side effects (diarrhea, rectal bleeding, tenesmus and occlusion) associated with high morbidity and mortality [2].

After irradiation, the death of proliferating stem cells, microvascular apoptosis and local ischemia can disrupt epithelium renewal. Moreover, in the gut, impairment of the epithelial barrier may result in increased penetration and absorption of toxic and immunogenic factors, leading to an uncontrolled immune response and a homeostatic imbalance in the epithelium. Disturbances to the repair process can lead to loss of tissue (ulceration) or pathological healing (fibrosis, fistula). Thus, rapid resealing of the epithelial surface barrier following injury is essential for preserving normal homeostasis. The intestinal epithelium is maintained by an intricate cell-replacement process in which terminally differentiated epithelial cells are continuously and rapidly replaced by the replication and differentiation of clonogenic epithelial cells located within the crypts. The recent identification of intestinal stem cell (ISC) markers such as *Lgr5* (leucine-rich repeat containing G protein-coupled receptor 5), *Sox9*, *Tert* (telomerase reverse transcriptase) and *Bmi1*, has made it possible to distinguish two functionally distinct ISC populations during homeostasis and the injury repair process. *Lgr5*+, *Sox9*+mark the rapidly dividing cells and identify a population of cells able to form *in vitro* organoids [3]–[5]. In support of Potten’s initial hypothesis, the ISC field has recently showed evidence of the presence in the intestine of *Sox9*+, *Bmi1*+and *mTert*+slow-cycling cells playing an important role in the regenerative process [6]–[9].

To minimize radiation damage to the intestine, several pharmaceutical strategies have been considered, based on the protection/suppression of the destructive process (Trefoil factor 3, Glucagon-like peptide-2) or stimulation of the regenerative process (Interleukin 11, Keratinocyte Growth Factor and R-SPONDIN). However, their efficacy is limited and further research is needed to investigate their safety and efficacy in patients. To date there are no effective Food and Drug Administration-approved therapeutic agents for significantly improving intestinal radiation-induced lesions [10]. Stem cell-based approaches using MSC have proved promising for the development of future therapeutic approaches. In mouse models of inflammatory bowel disease, MSC injection reduces the severity of colitis, prevents recurrence of the disease and reduces animal mortality [11]. In clinical trials, intravenously-injected MSC induce therapeutic benefits in patients with graft-versus-host disease suffering from gastrointestinal disorders such as peritonitis [12]. A phase II Crohn’s disease study also yielded promising efficacy in the treatment of rectovaginal and perianal fistulas [13]. Moreover, clinical grade expanded-MSC were strictly verified [14] and long-term follow-up of patients undergoing MSC administration affirms the safety profile of the treatment with no evidence of neoplastic structures [15].

In spite of the improvement of radiotherapy techniques that aim to target prostate, bladder or uterine tumors, the colorectum is located in the irradiation field as well. Therefore, the colorectum may be injured and damaged during pelvic radiotherapy protocols leading to organ dysfunction and colonic complications. In this study, we analyzed the therapeutic potential of MSC treatment on rat colonic epithelium in a context of radiation-induced ulceration. Previous work in our laboratory in rats showed no changes in colonic histology after fractionated colorectal radiotherapy [16], 26 weeks after the last dose of irradiation. Therefore, in this study, in order to obtain severe and irreversible damage a single high dose of irradiation (27Gy) centered on the rat’s distal colon was applied. This experimental model generates severe radiation-induced epithelial alterations histologically similar to those seen in patients treated with radiotherapy and who develop colorectal complications. Our results report proof of principle of MSC’s ability to reduce radiation-induced colonic epithelium ulceration. In this context, we also analyzed, *in vitro* and *in vivo,* the involvement of molecular signaling pathways on epithelial cell regulation after MSC treatment.

## Materials and Methods

### Animals, Irradiation, MSC Injection Protocol and Sample Collection

All experiments were performed in compliance with French laws and guidelines for animal experiments (Act no.92–333 of 2 October 2009) and approved by the Ethics Committee of Animal Experimentation “CEEA number 81″ (Protocol numbers: P07–15 and P07–16). The 300g wild-type male Sprague-Dawley (SD) rats were purchased from Charles River Laboratories (France). Animals were housed in double decker cages, three to a cage, with full access to food and water and light and dark cycles. All efforts are made to minimize suffering and all experiments are performed on anesthetized animals (TEM, anesthesia, Limoges, France) by isoflurane inhalation (AErrane, Baxter SA, Lessiness, Belgium). Animals were anesthetized and a single 27Gy dose was delivered by a ^60^Co source through a 2×3 cm window centered on the colorectal region. This configuration of irradiation also induces the irradiation of other organs located close to the colon as bladder, prostate or seminal vesicles. This single dose irradiation methodology, though it is not a model for human radiotherapy (fractionated irradiation), provides a good colonic ulcerative match for patients subjected to pelvic radiotherapy and who develop gastrointestinal complications. Right after irradiation (preventive protocol) or three weeks after irradiation then every two weeks (curative and iterative protocol), 5 million MSC were injected in the tail vein of the anesthetized rat. Animal behavior was monitored daily and suffering animals were euthanized. Euthanasia is performed by excess of anesthetic product. Colonoscopy analyses were done at 18 weeks on anesthetized rats with pediatric bronchoscope (Pentax, France).

### MSC Isolation, Characterization and Culture

MSC bone marrow was obtained by flushing femurs of seven-week-old rats ethically euthanized as previously described in the literature [17]. After ten days, the monolayer of adherent cells (P0) was seeded at 5,000 cells per cm^2^ (passage P1). At each passage the phenotype of amplified MSC was verified by flow cytometry using FACSort (BD Biosciences). Cells were incubated for 20 min at 4°C with phycoerytrin-conjugated mouse monoclonal antibodies against rat antigens. The percentage of CD90^+^(clone OX-7; BD Biosciences) and CD73^+^(clone 5F/B9; BD Biosciences) cells was analyzed and the absence of hematopoietic cells was verified with CD34 (clone ICO115, Santa Cruz) and CD45 (clone OX-1; Becton Dickinson, France) markers. On average, MSC expressed 94.8% CD90 (+/−3.3), 81.25% CD73 (+/−8.12), 2.13% CD34 (+/−0.79) and 6.4% CD45 (+/−1.15). Identical isotope antibodies served as controls. The potential of adipogenic, osteogenic and chondrogenic differentiation was also evaluated as described by Rochefort et al [17]. The abilities to form colony-forming unit fibroblasts (CFU-F) were also analyzed. Bone marrow total cells or peripheral blood mononuclear cells (after ficoll) were plated in triplicate at densities of 5×10^6^ cells per 25 cm^2^ or 15×10^6^ cells per 25 cm^2^, respectively. CFU-Fs were stained with violet crystal and counted after 10 days. For MSC conditioned medium reparation, MSC were seeded at 2×10^6^ cells in a 75 cm^2^ flask and cultured overnight in media without FCS. Then the supernatant was collected and frozen and the cells were counted.

### Tissue Samples

For rat samples, on the day of euthanasia, cardiac puncture was performed under isoflurane anesthesia. Collected blood was transferred in EDTA sample collection tubes and centrifuged (2500rpm, 20min) to separate white blood cells from red cells and plasma for ELISA and CFU-F analysis. Colonic mucosa was separated from muscularis propria by gentle dissection. Half of the sampling was frozen in liquid nitrogen for RNA preparation and the remaining tissues was used to prepare mucosa protein extracts by tissue disruption in PBS with protease inhibitors (complete Mini, Roche) and stored at −80°C until use. For histological analysis, the colorectum was fixed in 4% formaldehyde and embedded in paraffin. Human tissues were obtained according to institutional ethical guidelines (Gustave Roussy Institute) and French Medical Research Council guidelines. Tissue samples were obtained from tiny pieces of colon removed surgically from patients treated for rectal adenocarcinoma with preoperative radiotherapy (a total of 45Gy, delivered in fractions of 1.8 or 2Gy). Patients were notified that resections would be sent to pathologists for analysis but no ethics committee was consulted for operating waste. Nevertheless, with the laws (articles L1245-2, L1211-3, L1211-4, L1211-5, L1211-6 and LA211-7 of the Public Health Code) the surgical residues were subjected to the principles and rules in relation to the donation and procurement of human tissues. Surgery resection was done 5 to 7 weeks post radiotherapy. Slides were stained with hematoxylin-eosin-saffron. Tissues samples were numbered and anonymously analyzed.

### Histology, lesion Analyses and Immunohistochemistry (IHC)

Paraffin embedded colons were cut into circular sections of 5 µm and stained with hematoxylin-eosin-saffron (HES). The severity of colorectal damage was assessed using the radiation injury score modified from Langberg et *al*. A variable of the injury score was mucosal damage (ulceration, epithelial atypia and regeneration capacities), colitis *cystica profunda*, vascular sclerosis, fibrosis, muscular dystrophy and serosal thickening. Graduation of the injury was 0 = null; 1 = slight; 2 = moderate and 3 = severe. For immunohistochemistry, sections were deparaffinized and hydrated. For GFP (Green Fluorescent Protein) IHC, tissue sections were treated with 0.1% triton X-100 (Sigma-Aldrich) in PBS 1x (Gibco-BRL) at room temperature (RT) for 10 min. Then endogenous peroxidases were inhibited by incubation with 3% H2O2 in methanol at RT for 10 min. After saturation (X0909, DakoCytomation) rabbit anti-GFP diluted to 1/200 (Anaspec; 29779) was applied to the section for 1h at 37°C. The tissue sections were incubated with Envision kit anti-rabbit HRP (K4002; DakoCytomation) for 30 min at RT. For β-catenin and SOX9 immunostaining, tissue sections were placed in an antigen retrieval solution (0.01M citrate buffer, pH = 6 (DakoCytomation) for 3×5 min at 350W) and quenched for endogenous peroxidases as described above. After saturation (X0909, DakoCytomation) rabbit anti β-catenin at 200 µg/ml (ab2365; Abcam) or anti-SOX9 at 200 µg/ml (Sc-20095; Santa-Cruz) was applied to the section for 2h at RT. The tissue sections were incubated with Envision kit anti-rabbit HRP (K4002; DakoCytomation) for 30 min at RT. For PCNA (Proliferating Cell Nuclear Antigen) and WNT4 IHC, tissue sections were treated with 0.1% triton X-100 (Sigma-Aldrich) in PBS 1x (Gibco-BRL) at RT for 10 min. Then endogenous peroxidases were inhibited by incubation with 3% H2O2 in methanol at RT for 10 min and placed in an antigen retrieval solution (0.01M citrate buffer, pH = 6 (DakoCytomation)) for 3×5 min at 350W. After saturation (X0909, DakoCytomation) mouse anti-PCNA used at 525 µg/ml (clone PC10; DakoCytomation) or rabbit anti-WNT4 used at 1 mg/ml (Lifespan Biosciences; LS-C112550) was applied to the section for 1h at 37°C. The tissue sections were incubated with kit LSAB2 (K0609; Dakocytomation) or Envision kit anti-rabbit HRP (K4002; DakoCytomation). Staining was developed with Histogreen substrate (E109; Abcys). Sections were counterstained with Fast nuclear red (H-3403; Vector), dehydrated and mounted. Isotype control antibodies are used as negative controls. Immunohistochemistry analyses were performed on n = 8 animals.

### Intestinal Cell Culture Inhibition Conditions and Immunofluorescence

IEC18 were obtained from ATCC (ref ATCC-CRL-1589, Manassas, VA) and cultured in DMEM, high glucose (Invitrogen) supplemented with 5% FCS, 1% Penicillin Streptomycin and 1% Glutamine (Gibco-BRL; Invitrogen). Cells were seeded at 60,000 cells/well in 6-well plates, starved for 24 h without FCS, irradiated or not at 15Gy with a ^137^Cs source (1,1 gy/min). Right after irradiation, MSC conditioned medium resulting from 3 to 5 different bone marrow expansions or medium alone was applied to the cells. IEC18 were treated or not with different inhibitors: the PI3-kinase (Phosphoinositide 3-kinase) inhibitor (LY-294002), used at final concentration of 10 µg/ml, was obtained from Promega (Madison, USA), recombinant DKK1 (Dickkopf Homolog-1) was obtained from R&D systems (UK) and used at 0.4 µg/ml, MEK1 (MAP Kinase kinase) inhibitor (PD98059) was obtained from Invitrogen and used at 25 µg/ml and JAK1 (Janus Kinase-2) (33 µM) and Casein kinase I inhibitors (33 µM) were obtained from Calbiochem (Merck KGaA, Darmstadt, Germany), CAMKII (calmodulin-dependent protein kinases II) used at 4 µM (KN-93) and PKC (Protein Kinase C) used at 1.5 µM (RO 31-8220) from Sigma-Aldrich. After 48 hours, supernatants were collected for protein analysis and IEC18 were trypsined, numbered and frozen for PCR. Each condition was realized in triplicate. For immunofluorescence, IEC18 were seeded on slides and cultured until 70% of confluence. After washing in PBS1x, cells were fixed in methanol then permeabilized in Triton X100 and saturated in 5% goat serum for 20 min at RT. Slides were incubated with rabbit anti β-catenin antibody diluted to 1/1000 (ab2365; Abcam) then goat anti-rabbit Alexa 568 (A–11036, Invitrogen) diluted to 1/200. Then slides were incubated with DAPI and Vectashield Hard Set (H–1500, Vector), kept 15 min at 4°C to polymerize and visualized under microscope (Leica, France).

### Protein Measurement, ELISA and Western Blot

Protein measurement was done using a BCA assay kit (Sigma, QuantiPro BCA assay kit ref QPBCA-1kt). Quantitative determination of EGF (Epithelial Growth Factor), SDF-1 (Stroma derived factor-1alpha) (R&D System), HGF (Fibroblast Growth Factor), KGF, IGF2, IGF1 (Insulin Growth Factor 1 and 2), IL11 and βFGF (UsnLifeScience Inc, China) in plasma, supernatant or mucosae extracts were performed using ELISA, according to the manufacturer’s recommendations. ELISA were performed on n = 6 animals per group. Analysis of C-JUN phosphorylation was done using western blot. Eight micrograms of proteins from IEC 18 cell lysate (from 3 different IEC18 cultures loaded in triplicate) were separated by SDS-polyacrylamide gel electrophoresis before transfer onto nitrocellulose membrane. Membranes were blotted with rabbit anti PHOSPHO-C-JUN (sc-16312-R; Santa Cruz) and anti C-JUN (sc-45; Santa Cruz) the primary antibodies diluted to 1/200ème. Then the secondary antibody HRP conjugated (Amersham, France) directed against rabbit antigens were applied to the membrane diluted to 1/2000ème. Blots were developed using the enhanced chemioluminescence method (Millipore, France). Blot quantification was done with a BIORAD scanner (GS-800 calibrated densitometer) and Quantity One 4.5.2 software.

### RNA Isolation, Reverse Transcription and Real-time PCR

Tissue and cell total RNA was prepared with RNeasy mini kit (Qiagen, France) and cDNA were obtained with the High Capacity Reverse Transcriptase cDNA kit (Applied Biosystems). Real-time quantitative PCR was performed using Taqman gene expression assays (Applied Biosystems). The samples (n = 6 for each group) were loaded in duplicate and fold changes were calculated using ΔΔCt normalizing to *gapdh.*

### Statistical Analysis

Results are expressed as mean ± SEM (standard error of the mean). Results were compared between groups by a t-test or a one-way ANOVA followed by a Tukey test using Sigmastat software (Systat Software Incorporation, GmbH, Erkrath, Germany). A value of p≤0.05 was considered to be statistically significant.

## Results

Histopathologic analysis of colonic epithelia on human resections and rat tissues exposed to radiation.

**Figure 1:**
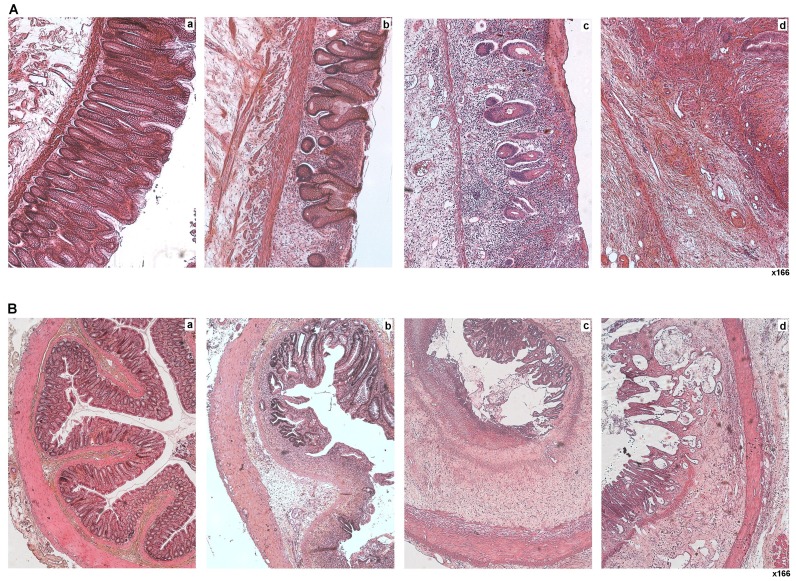
Characterization of radiation-induced colonic epithelium damage (A) Representative pictures of human colorectal lesions induced by preoperative radiotherapy (total of 45 Gy delivered in 2 Gy fractions) after 6 weeks in patients treated for adenocarcinoma. Colorectal tissues outside (a) or inside (b, c, d) of the irradiated zones. (B) Representative pictures of colorectal lesions induced in rats with 27Gy (single dose) of irradiation. (a) Control, (b) irradiation at 2, (c) 8 and (d) 21 weeks. Tissues are stained with hematoxylin-eosin-saffron (HES). Pictures were taken on tissues located inside the irradiated field. Original magnification, x166. Colorectal irradiation leads to characteristic lesions that can be separated into dystrophic (A b and B b,c,d) and fibronecrotic zones (A c,d and B c,d).

Histologic examination of normal human mucosa revealed numerous organized crypts lining dense muscularis mucosa (Figure 1A,a). In the irradiated field, characteristic damage is observed, which can be separated into dystrophic and fibronecrotic zones. In dystrophic areas, mucosal lesions consist of atypical crypts and edema (Figure 1A,b). In fibronecrotic areas, crypts are almost completely absent and mucosa is replaced by intense inflammatory cell infiltrate (Figure 1A,c). Non-healing mucosal ulcers are usually associated with fibrosis, which affects the mucosa and sub-mucosa with dense extracellular matrix deposition (Figure 1A,d). In our experimental model of rats subjected to colorectal irradiation, histopathological lesions observed in the irradiated field were similar to those seen clinically. We observed apoptotic crypts and sub-mucosal edema one week after irradiation. At two weeks, we observed dystrophic zones or fibronecrotic zones. Substantial sub-mucosal edema was also visible (Figure 1B,b). Eight and 21 weeks after irradiation, fibronecrotic areas in mucosa and sub-mucosa were also observed (Figure 1B,c -1B,d). While atypical crypts usually described as highly dividing cells could still be seen in the lamina propria and the sub-mucosa, there was a worsening of the lesion leading to transmural fibrosis associated with vascular sclerosis and dystrophy of the muscularis propria (Figure 1B,d).

### MSC Engraft in Colonic Mucosa and Improve Endogenous MSC Mobilization into Blood

**Figure 2:**
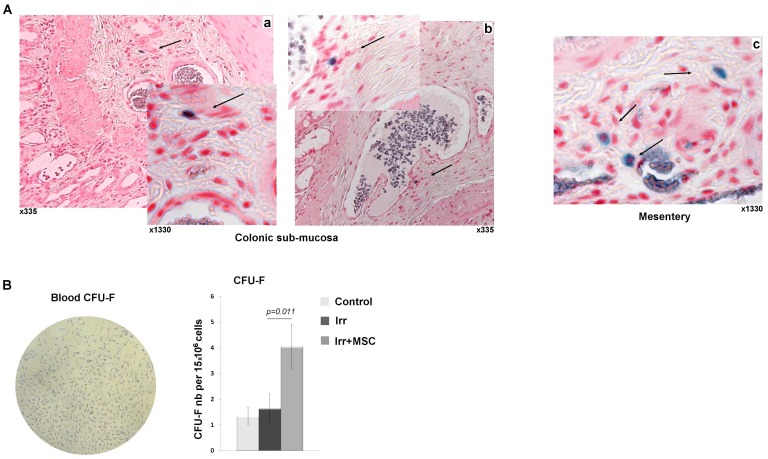
MSC engraft in colonic mucosa and improve endogenous MSC mobilization into blood. (A) Intravenous injected MSC engraft in the colon. Detection of GFP-MSC (black arrow) in irradiated (a, b) colonic sub-mucosa or (c) mesentery, 1 week after injection using GFP-specific antibodies. Original magnification, x335 and x1330. (B) Mobilization of endogenous MSC in blood induced by MSC treatment. Representative picture of MSC morphology in blood CFU-F and quantification of CFU-F number per 15×10^6^ plated cells. MSC treatment increases the number of blood-derived CFU-F (n = 11 for each group). Results are expressed as mean ±SEM and compared between groups by one-way ANOVA followed by a Tukey test.

MSC were prepared from the bone marrow of green fluorescent protein (GFP)-transgenic SD rats, then verified for GFP expression and injected intravenously in immunocompetent SD rats immediately after colorectal irradiation. We analyzed MSC engraftment on colonic sections taken every 1500 µm throughout the distal colon. Experiments revealed the presence of GFP cells in the sub-mucosa and in the mesentery, near the vessels, until one week after MSC injection (Figure 2A). However, GFP-MSC injected could not be detected after two weeks. We then tested the ability of MSC therapy to induce endogenous MSC mobilization in the blood. It has been previously described that endogenous MSC can be mobilized from storage organs into blood in response to tissue damage [17]. These MSC-mobilized cells could participate in tissue regeneration [18]. To quantify MSC frequency in peripheral blood, we used their ability to form colony forming unit-fibroblasts (CFU-F) in culture. The morphology of blood-derived CFU-F was similar to those obtained from bone marrow. Quantitative analysis showed a 2.5-fold (p = 0.011) increase in the number of blood-derived CFU-F, three days after MSC treatment, in comparison to the irradiated group (Figure 2B). As CFU-F were GFP-negative, we can exclude the presence of injected cells in blood three days after the treatment. At the same time, we quantified the level of chemoattractant molecule SDF-1α in plasma by ELISA. We detected 695.6±57.9 pg/ml in the irradiated group and 1161.2±91.1 pg/ml in irradiated and MSC treated group, i.e. a 1.7-fold (p<0.05) increase induced after MSC treatment. These results suggest that mobilization of endogenous MSC into the bloodstream induced by MSC-based therapy could be at least stimulated by the secretion of paracrine factor such as SDF-1α.

### MSC Therapy Decreases Radiation-induced Colonic Ulcers

**Figure 3:**
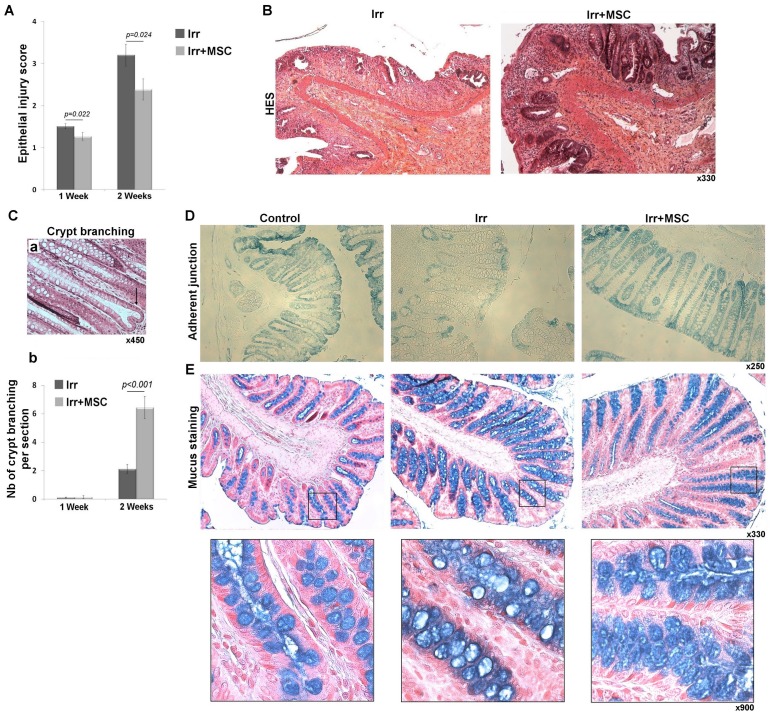
MSC treatment reduces colonic epithelial lesions. (A) Epithelial injury score. (B) Representative histological pictures of atypical crypts 2 weeks after irradiation and irradiation combined with MSC treatment in the ulcerated zone. Original magnification, x330. (C) MSC therapy increases the number of crypt branching per circular section 2 weeks after irradiation. In A and C results are expressed as mean ±SEM and compared between groups by t-test. (D) Representative pictures of adherent junction staining by immunohistochemistry using anti-β-catenin antibodies (blue staining without counterstaining, original magnification, x250) and (E) mucus staining by alcian blue coloration 2 weeks after irradiation in the healthy margins of the colon. Original magnification, x330 and x900. Functional parameter modifications of the colonic epithelium induced by irradiation were not observed after MSC treatment. All analyses were performed on tissues located inside the irradiated field. In all experiments n = 8 for each group.

Quantification of the therapeutic potential of MSC infusion on epithelial injury induced by ionizing radiation was analyzed on histological sections using HES coloration. All analyses were performed in the irradiated field. 5×10^6^ MSC were injected right after irradiation and results demonstrated statistically significant improvements in the epithelial injury score at one and two weeks of 16.05% (p = 0.022) and 25.48% (p = 0.024), respectively (Figure 3A). This therapeutic benefit is cell dose-dependent; the injection of lower number of MSC (1×10^6^ and 0.1×10^6^) displayed no significant benefit on the radiation-induced epithelial injury score and injection of higher number of MSC (10×10^6^) did not further increase the benefit (data not shown). Within the ulcerated area, we observed a characteristic benefit of MSC therapy at two weeks with improvement of the compensatory re-epithelization process that originates in atypical crypts with actively dividing epithelial cells (Figure 3B). Newly-formed crypts are important for the epithelial regeneration process in the intestine and can be achieved through crypt branching (Figure 3C,a). While the underlying mechanisms of crypt branching are unknown, it has been suggested that crypt size is important in initiating this phenomenon. We used morphometric analysis to evaluate crypt depth and counted the number of crypt branching per transversal section located near the ulcerated zone. At two weeks, we observed increased crypt size in irradiated rats (363.07±5.7 µm) compared to controls (266.4±2.9 µm), which was even greater in the irradiated, MSC-treated group (475.91±8.9 µm). This crypt size increase is associated with a higher number of crypt branching which is substantially higher (p<0.001) in irradiated animals infused with MSC compared to irradiated animals not treated with MSC (Figure 3C,b). The number of crypts per transversal section was also higher (p<0.001) in the irradiated, MSC-treated group compared to the irradiated group (data not shown). After irradiation, we also observed at the margin of the ulcerated areas edematous areas where crypt structure was preserved. We reported functional parameter modifications in this zone, which are not observed after MSC treatment. Immunostaining experiments using anti β-catenin antibodies reveal a decrease in adherent junction protein expression after irradiation, while this level of expression is similar to the control after MSC treatment (Figure 3D). Furthermore, a radiation-induced reduction of goblet cell content (i.e. acid mucus stained in goblet cells with alcian blue) was not reported after MSC treatment (Figure 3E). Altogether, these results demonstrate that infused MSC reduce radiation-induced ulcers by improving the regenerative process not only in ulcerated areas but also in the ulcer margins.

### MSC-secreted Molecules Increase the Number of Crypt Epithelial Cells: Involvement of the non-canonical WNT Pathways

**Figure 4:**
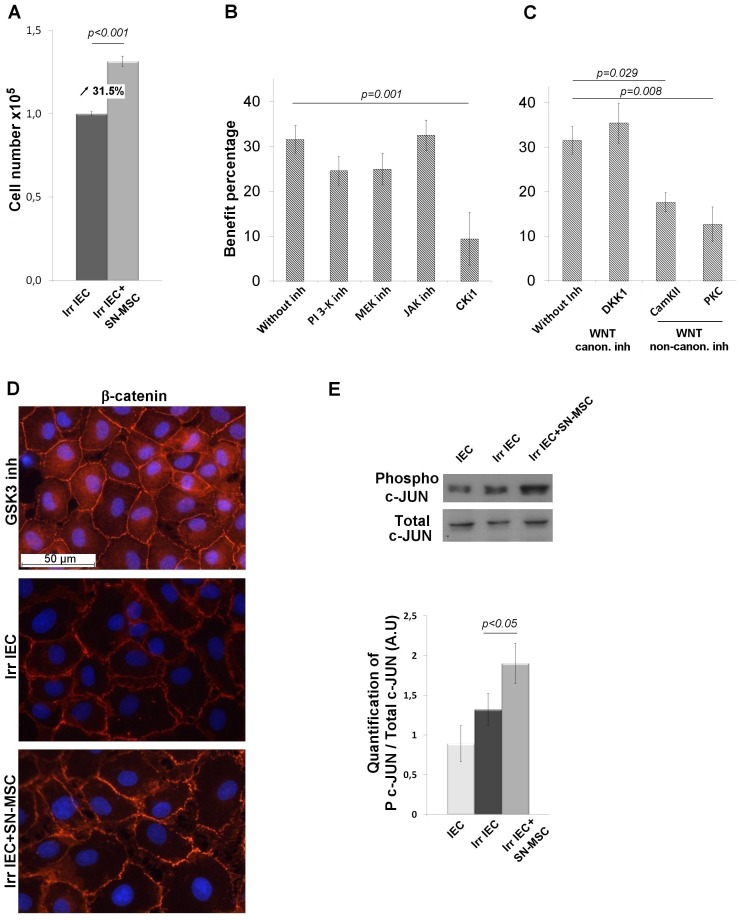
*In vitro* analysis of paracrine mechanisms of MSC action. (A) MSC supernatant increases the number of IEC-18 after irradiation. Data are the mean of 9 independent experiments performed in triplicate. (B) Inhibition of AKT (Ly294002), MEK (PD-98059), JAK1 and WNT (CK1i) signaling pathways using specific blocking agents. (C) Inhibition of the canonical (DKK1) and non-canonical (KN-93 and RO31-8220) WNT pathways. Results are expressed as percentage of MSC benefit (Irradiated/Irradiated with SN-MSC x100). In A, B and C results are expressed as mean ±SEM and compared between groups by t-test. (D) Representative immunofluorescence experiment to visualize β-catenin nuclear translocation in irradiated IEC-18. (E) Representative western blot using antibodies against phosphorylated c-JUN or total c-JUN. The ratio of phosphorylated c-JUN/total c-JUN analyzed on irradiated IEC18 is increased after SN-MSC incubation. Results are expressed as mean+/−SEM of 4 independent experiments and compared between groups by one-way ANOVA followed by a Tukey test.

To analyze paracrine mechanisms of MSC action and signaling pathway involvement in the re-epithelization process, we performed *in vitro* experiments using irradiated, non-transformed rat crypt epithelial cells (IEC-18) cultured or not with MSC-supernatant (SN-MSC). In our culture conditions, rat MSC express βFGF, HGF, KGF, IL11, *R-spondin*, *wnt2*, *4*, *5* and *11* molecules, factors described as facilitating intestinal mucosal repair. We also observed that SN-MSC increases the expression of βFGF, KGF, IL11 and *wnt4* by IEC-18 (data not shown). After irradiation we observed a decrease of the number of IEC-18 (2.9 10^6^±0.9 for IEC-18 and 1.0 10^6^±0.1 for irradiated IEC-18). We reported a 31.55% increase (p<0.001) in the number of irradiated IEC-18 in the presence of SN-MSC (Figure 4A). Blocking PI3-K, MEK or JAK signaling pathways decreases the number of irradiated IEC18 but does not significantly modify the MSC-induced benefit (Figure 4B). Although the three inhibitors were added at the same time to the culture medium, SN-MSC increases the number of irradiated IEC-18 (data not shown), demonstrating the non-redundant role of PI3-K, MEK and JAK pathways. Nevertheless, blocking WNT signaling pathways with CK1i cancelled out the benefit of SN-MSC (Figure 4B). The use of DKK1, which specifically blocks the canonical WNT pathway, did not affect the benefit of SN-MSC (Figure 4C). The canonical WNT pathway is dependent on the stabilization of β-catenin and its translocation to the nucleus. We validated the absence of this pathway involvement in analyzing β-catenin localization by means of immunofluorescence on irradiated IEC-18. As GSK3 inhibitor induces stabilization of intra-cellular β-catenin in irradiated IEC-18, incubation with SN-MSC maintains β-catenin localized to the cell membrane in irradiated IEC-18 (Figure 4D). Non-canonical WNT pathways are the planar cell polarity (PCP) pathway and the calcium-dependent pathway (WNT/Ca^2+^). Using specific inhibitors of these pathways, we demonstrated significant loss of the benefit provided by SN-MSC (Fig. 4C). Downstream signalization of non-canonical pathways induces c-JUN phosphorylation. Western blotting analysis revealed an increase (p<0.05) of c-JUN phosphorylation in irradiated IEC-18 after SN-MSC incubation (Figure 4E). These *in vitro* results demonstrate that MSC, via paracrine mechanisms, increase the number of crypt epithelial cells through the non-canonical WNT pathways.

### After Colorectal Irradiation, MSC Therapy Induces the Proliferation of Crypt Epithelial Cells and Increases WNT4 Expression by Epithelial Cells

**Figure 5:**
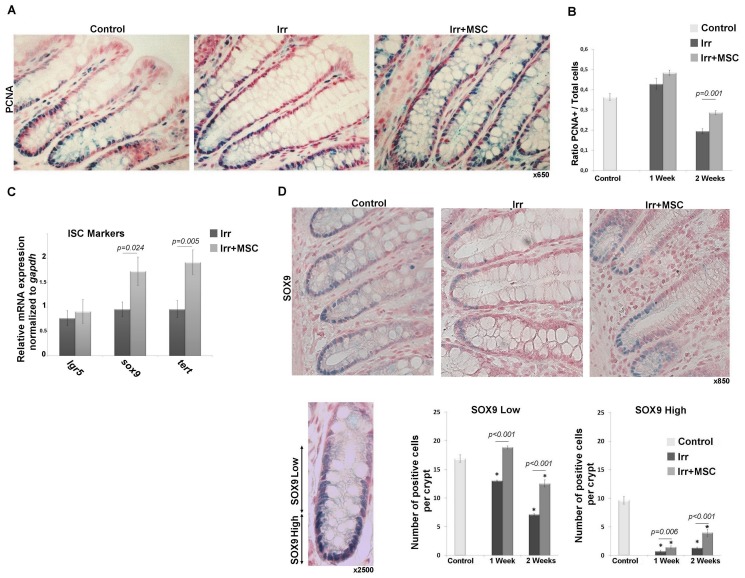
MSC treatment stimulates epithelial proliferation and increases the number of Sox9-high positive progenitor/stem cells. (A) *In vivo* analyses of epithelial proliferation. Representative pictures of PCNA immunostaining (blue staining) 2 weeks after irradiation. Original magnification, x650 (B) Quantification of PCNA-positive cells per total number of crypt cells. Data were compared between groups by one-way ANOVA followed by a Tukey test. (C) Relative mRNA expression of ISC markers in colonic mucosa of irradiated and irradiated MSC-treated rats. Results were normalized to *gapdh* housekeeping gene and standardized to control level (control expression = 1). Comparison between groups was evaluated by t-test. (D) Representative pictures of SOX9 immunostaining (blue staining) 2 weeks after irradiation. Original magnification, x850. Variation of SOX9 expression levels in rat colonic crypt and quantification of SOX9-low and SOX9-high -expressing cells by crypt. All analyses were performed on tissues located inside the irradiated field. Results were compared between groups by one-way ANOVA followed by a Tukey test. *p<0.001 *versus* control groups. In B no statistically differences were observed between 1 week and 2****weeks control groups, thus control groups were pooled. In A, B and D experiments n = 8 for each groups. In C experiments n = 6 for each group.

**Figure 6:**
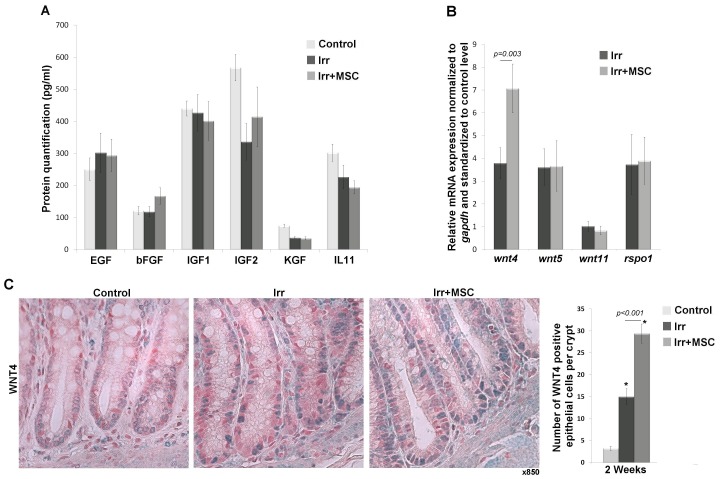
*In vivo* expression of molecular factors involved in the proliferation of epithelial cells 2 weeks after irradiation. Total proteins and RNA were extracted from irradiated colonic mucosa of different groups and analyzed by (A) ELISA or (B) RT-PCR, respectively. Relative mRNA expression of *wnt*-related genes were normalized to *gapdh* housekeeping gene and standardized to control level (control expression = 1). Expression of *wnt-1, 2* and *3* was not detected in any of the 3 groups. *Wnt4* expression increased by 2-fold after irradiation and 4-fold after irradiation and MSC treatment. Results were compared using t-test. (C) Representative pictures of WNT4 immunostaining (blue staining, original magnification, x850) and quantification of the number of WNT4 expressing epithelial cells per crypt 2****weeks after irradiation. WNT4 immunostaining in epithelial cells increased after irradiation and increased even more after MSC treatment. Results were compared between groups by one-way ANOVA followed by a Tukey test. *p<0.001 *versus* control groups. In A, B, and C experiments n = 6 for each groups.

Epithelial proliferation was assessed by counting PCNA-positive cells per total cells of the crypt on sections of the colon adjacent to the ulcer. Two weeks after irradiation, the number of proliferating cells decreased compared to the control group. At this time, the increased proliferation observed in the irradiated and MSC-treated group compared to the irradiated group is 32.1% (Figure 5A and B). We also demonstrated through gene expression analysis in irradiated colonic mucosa that MSC treatment increases *sox9* and *tert* ISC markers (Figure 5C). Immunohistochemistry analyses using the SOX9 marker (Figure 5D) enabled us to distinguish low-expressing cells (described as transit-amplifying progenitor cell zone) and high-expressing cells (described as cells with stemness characteristics). As already demonstrated in the colon [5] we found a majority of proliferating cells that also express SOX9 at the bottom of the crypt. One and two weeks after irradiation, we observed a decrease in SOX9+cell numbers. The reduction is moderate for SOX9-low cells and drastic for SOX9-high cells. MSC treatment in irradiated rats increases the number of SOX9-low cells compared to values obtained in irradiated animals (x1.44 at one week (p<0.001) and x1.48 at two weeks (p<0.001)). MSC treatment also limits radiation-induced reduction of the number of SOX9-high cells and the major effect (p<0.001) is detected at two weeks (x4.5 in irradiated and MSC treated rats *versus* irradiated rats). To evaluate molecules involved in crypt-cell proliferation, we performed *in vivo* analysis of growth factor secretion (EGF, βFGF, IGF1/2, KGF and IL11), *r-spondin* and *wnt* gene expression (*wnt2*, *3*, *4*, *5*, *6* and *11*) on colonic mucosa (Figure 6 A, B). Except for *wnt4* gene expression, results do not show differences between irradiated and irradiated MSC-treated animals, in accordance with our *in vitro* findings. Quantification of *wnt4* expression using RT-PCR demonstrated an increase in its expression after irradiation by 2-fold, and an even higher increase by 4-fold (p = 0.003) after irradiation and MSC treatment (Figure 6B). This result was confirmed at the protein level by WNT4 immunostaining on colonic slides (Figure 6C). We observed that the number of epithelial cells expressing WNT4 increased after irradiation and increased even more after MSC treatment (Figure 6C). Our results demonstrate MSC’s *in vivo* ability to maintain regenerative properties by stimulation of proliferating colonic epithelial cells. This effect might be potentiated by epithelial cell autocrine secretion of the non-canonical WNT4 factor.

### Analysis of the Therapeutic Efficacy of MSC on Established Radiation-induced Damage

**Figure 7:**
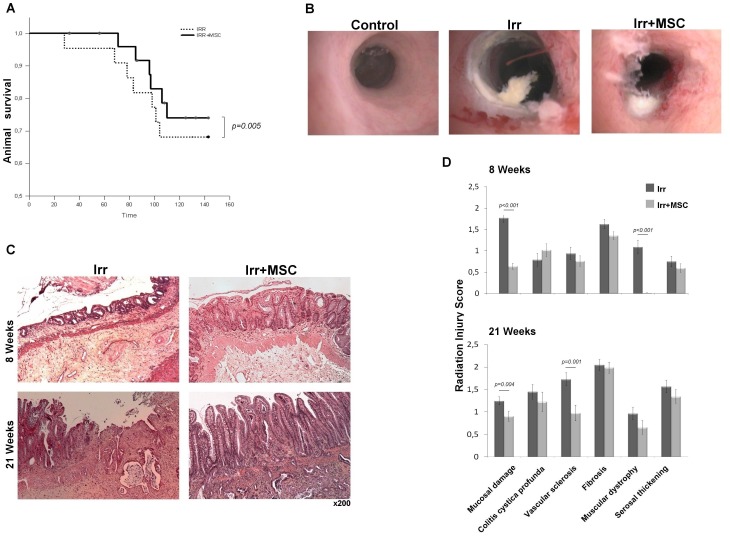
Therapeutic benefit of iterative injections of MSC on established colon damage induced by ionizing radiation (first injection performed 3 weeks after irradiation, then every 2 weeks). (A) Comparison of animal survival between irradiated (n = 25) and irradiated, MSC-treated group (n = 20); *p* value determined by log RANK test. (B) Representative endoscopy pictures 18****weeks after irradiation (n = 5 for each group). Profound ulcerative area with white necrotic tissue is observed in irradiated animals. In MSC-treated rats deep necrotic areas are less extensive. (C) Representative epithelial damage visualized by HES coloration 8 and 21****weeks after irradiation. Analyses were performed on tissues located inside the irradiated field. f. Original magnification, x200 (D) Radiation injury score 8 and 21****weeks after irradiation (n = 6 for each group). Data were compared by t-test.

**Figure 8:**
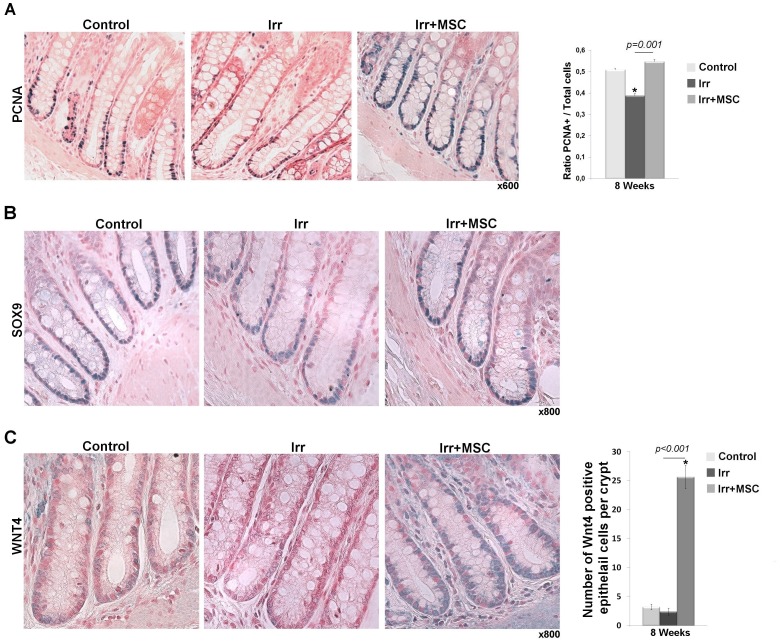
Iterative injection of MSC on established radiation-induced colic ulcer increases the ability of epithelial cells to proliferate. This effect is associated with an increase in SOX9 positive cells and a boosted WNT4 molecule expression by epithelial cells. (A) Representative pictures of PCNA immunostaining (blue staining) 8 weeks after irradiation and quantification of PCNA-positive cells per total number of crypt cells. Original magnification, x600. Irradiation reduces the proliferative ability of epithelial cells while the proliferative process is maintained at a basal level after MSC treatment. (B) Representative SOX9 immunostaining 8 weeks after irradiation. Irradiation drastically reduces the number of SOX9-high cells that are restored after MSC treatment. (C) Representative pictures of WNT4 immunostaining in colonic mucosa and quantification of the number of WNT4 expressing epithelial cells per crypt. Eight weeks after irradiation, WNT4 expression was not different from controls in all analyzed rats, while WNT4 expression in colonic mucosa of MSC treated rats was higher than in the control group. All analyses were performed on tissues located inside the irradiated field. In all experiments n = 6 for each group. Results were compared between groups by one-way ANOVA followed by a Tukey test. *p<0.001 *versus* control groups. Original magnification, x800.

Three weeks after irradiation (as already described for two weeks) glandular recovering (atypical crypt regeneration) alternates with profound ulcerated areas. Iterative injections of MSC starting at this time increase (p = 0.005) animal survival (Figure 7A). Colonic lesions were also studied by endoscopy. Representative pictures are presented in Figure 7B. We observed profound ulcerated areas with white necrotic tissues in irradiated rats. Hemorrhages and petechial vessels were also observed in numerous irradiated animals. In MSC-treated rats, deep necrotic areas were less extensive and hemorrhages were less pronounced but petechial vessels were still observed. Scoring of lesions on HES slides demonstrated significant muscular and vascular improvement at eight and twenty-one weeks, respectively. The localized irradiation delivered to the colon induces a significant score of fibrosis and serosa thickening at eight and twenty-one weeks. This method of scoring did not enable us to highlight statistical improvement of these parameters by MSC treatment. However, we observed mucosal improvement at both times following MSC treatment (Figure 7C, D). Mucosal scoring includes epithelial atypia, re-epithelization ability. All of these criteria were significantly improved in the MSC-treated group compared to the non-treated group. As previously studied in a preventive protocol, we counted PCNA-positive cells per total cells of the crypt, on sections located at the margin of the ulcer. We demonstrated that iterative injections of MSC on established radiation-induced lesions restore the ability of crypt epithelial cells to proliferate, which is impaired eight weeks after irradiation (Figure 8A). This result is correlated with an increase of SOX9-high expressing epithelial cells in MSC-treated animals compare to irradiated animals (Figure 8B). We also evaluated WNT4 molecule expression in colic mucosa at the margin of the ulcer by immunohistochemistry (Figure 8C). The number of WNT4 expressing epithelial cells per crypt was counted and results show that WNT4 molecule expression is boosted in irradiated animals treated by MSC. These results cannot demonstrate reversion of fibronecrosis induced by radiation. Nevertheless, MSC treatment induces a therapeutic benefit on the colonic epithelium by reducing established ulceration through the stimulation of the regenerative process from ulcer margins.

## Discussion

The present findings demonstrate that a single dose of colorectal irradiation induces colonic epithelial alterations. In this irradiation methodology, other organs located close to the colon, like the bladder, the prostate or seminal vesicles may be in the irradiated field. Irradiation of these organs might induce time-dependent tissue damage such as what has already been described for the bladder [19]–[21]. Physiological cross-talk between different visceral organs is necessary to function in a coordinated manner [22]. The pathology of one visceral organ increases the inflammatory process in other organs and the development of cross-organ sensitization in the pelvis leading to functioning alterations [23]. Direct radiation effects on various organs associated with cross-organ sensitization might be at the origin of a worsening of colonic pathology. In our model, radiation toxicity leads to severe chronic mucosal ulceration and fibrosis. In previous studies performed using the same model, we showed that, in addition to colonic epithelial fibrosis, irradiation also induces inflammatory processes and vascular damage [24], [25]. These lesions seem to be similar to those observed in patients subjected to pre-operative radiotherapy. Using this model we demonstrated the therapeutic benefit of MSC on radiation-induced severe epithelial colonic ulceration. The therapeutic efficacy of MSC was observed at all stages of lesion development (i.e. in established or non-established lesions) at the time of cell treatment. This could be explained by the capacity of MSC to stimulate the regenerative process, which is initiated after irradiation but at a low level and with little efficacy. In all experimental protocols used in this study, we provided evidence that MSC therapy maintains the proliferative ability of epithelial cells positive for the SOX9 progenitor/stem cells marker, which are located in the ulcer margins. MSC treatment therefore improves colonic epithelium renewal. Our study postulates that MSC could act through paracrine mechanisms in which WNT4 might play a role in the stimulation of the regenerative process. This study constitutes a first approach to being able to argue in favor of the use of MSC in order to reduce irreversible, radiation-induced colonic ulceration.

MSC-mediated secretion of a broad range of bioactive molecules induces more significant biological effects than their ability to differentiate [26]. As already demonstrated by Lee et al, [27], in our model we also observed a high number of injected MSC trapped in the lung initially and until three days after their injection (data not shown). Lung-trapped MSC have been reported to improve myocardial infarction through the abscopal effect [27]. Such a mechanism could also be responsible for the initial benefit induced by MSC on colonic epithelial regeneration. In addition to the presence of MSC in the lung, we also observed transient engraftment of these cells in irradiated mucosa. Accumulation of evidence shows that MSC are also able to migrate into irradiated tissues after intravenous delivery [28]. It has been hypothesized that MSC will engraft in tissue through the same mechanism as leukocytes. Many of the molecules involved in the tethering, rolling, adhesion and transmigration of leukocytes from the bloodstream into the tissue are known to be expressed on MSC [29]. However, colorectal irradiation leads to a local inflammatory environment that is unfavorable to the survival of MSC. Thus, the secretion of regenerative factors by MSC engrafted in the colon was probably followed by their rapid removal. This could explain the common difficulty in highlighting MSC engraftment in damaged tissues. We also observed that MSC therapy increases plasmatic SDF-1α and demonstrated an increase of endogenous circulating MSC. SDF-1α plays an important role in the mobilization of MSC by down-regulating adhesion molecules that hold them in their niche [30]. Our study thus supports the idea that MSC therapy using secreted factors could enhance the pool of endogenous MSC by mobilizing them from organ storage. This endogenous MSC mobilization could indirectly ensure the continuation of tissue regeneration even if injected cells are partially lost. Mobilization of endogenous MSC to promote tissue repair has already been reported after tissue damage [17], [18] but never after MSC therapy, as was demonstrated in this study.

The intestinal epithelium undergoes rapid, continuous homeostatic renewal. Upon injury, the integrity of the intestinal mucosal surface is rapidly re-established because of the epithelium’s powerful regenerative capability. The ability of the host to respond to intestinal injury requires a highly orchestrated response involving migration, proliferation and differentiation of the epithelial cells from the ISCs. Our study demonstrates that radiation-induced reduction of colonic epithelial cells with progenitor or stemness characteristics (SOX9+) is limited by MSC therapy. MSC treatment of irradiated animals transiently maintains a basal number of SOX9-low cells (at one week) and also increases the SOX9-high cells, which are substantially reduced after irradiation. These effects are associated with an increase in the number of proliferating cells in the colon crypt. Recent studies have demonstrated that following *lgr5*+cell depletion (that are also SOX9-high) as a result of genetic mutation or irradiation, the number of slow-cycling cells (SOX9-low) increases, giving rise to *lgr5*-expressing cells [6]–[9]. Moreover, during the regenerative process following irradiation, SOX9-low slow-cycling cells acquire an *in vivo* proliferative potential and exhibit the ability to form organoids *in vitro*, whereas the same non-irradiated subset failed [9]. Altogether, results from various studies suggest that crypt regeneration involves the activation of a subset of low-proliferating cells that can adopt or dedifferentiate into a “stem cell-like” state [31] with a high proliferation capacity. Our results are in accordance with this concept and seem to demonstrate that after radiation injury, MSC therapy might improve this process.

As previously described for bone marrow hematopoietic stem cells [32], MSC might be an essential component of the intestinal epithelial stem cell niche, providing an optimal microenvironment for stem cell function. Mesenchymal-epithelial paracrine interactions involved in ISC maintenance and activation depend on a large array of signaling molecules. The WNT glycoprotein family is expressed specifically in the mesenchymal and epithelial compartments in the adult intestine [33] and the WNT pathways have emerged as a key regulator of maintaining epithelial homeostasis [34]. Our data support the assumption that MSC efficacy on the regenerative process of colonic epithelium after its irradiation may involve WNT signaling pathway activation through mesenchymal-epithelial paracrine interactions. The WNT glycoprotein family is a highly conserved ligand that acts through the canonical (WNT1, 2 and 3) or non-canonical (WNT4, 5a, 6 and 11) signaling pathways. Our *in vitro* experiments show that the inhibition of the canonical WNT pathway by DKK1 does not modify the increase of epithelial cells number induced by SN-MSC. Moreover, we observed that SN-MSC does not induce β-catenin nuclear translocation in colonic epithelial cells, usually observed after canonical WNT pathway activation. We can thus exclude the involvement of the canonical WNT pathway in MSC’s ability to induce epithelial proliferation in a context of radiation-induced alteration. Involvement of the WNT canonical pathway in epithelial proliferation along the gastrointestinal tract is controversial [35], [36]. Such discrepancies could be explained by the differential responsiveness of segments of the digestive tract to canonical WNT agonists/antagonists or to the relative abundance of WNT agonists in the colon [33]. This observation is in accordance to our results. Except for *r-spondin*, we cannot demonstrate the expression of the *wnt*-canonical gene in rat colonic mucosa. We clearly show *in vitro* an involvement of non-canonical WNT pathways in SN-MSC ability to increase epithelial cells and our results also suggest an autocrine regulation of the epithelial cells through WNT4 secretion. Our *in vivo* study points in the same direction. We thus demonstrated that after irradiation, MSC treatment stimulates epithelial proliferation and increases the number of SOX9-high positive cells associated with an increase in WNT4 expression by colonic epithelial cells. Altogether, these data support the involvement of the non-canonical WNT4 pathway in the ability of MSC to increase progenitor/stem cell activation and therefore to favor the epithelial regenerative process of the colon after radiation-induced epithelium ulceration. In regenerative medicine, it also been demonstrated in a hindlimb ischemia model that MSC-secreted WNT4, which increases with hypoxia, plays an essential role in vascular and skeletal muscle fiber regeneration [37]. Another study also demonstrated that MSC genetically engineered to express WNT4, enhance osteogenesis and improve the repair of craniofacial defects [38].

Canonical and non-canonical WNT pathways act via FRIZZLED family receptors and it has been demonstrated that LGR5 (R-SPONDIN receptor) associate with FRIZZLED in β-catenin signaling [39], reinforcing the notion that WNT signaling and stem cell biology are closely related [40]. Moreover, LGR5 can also act through the non-canonical signaling pathways depending on the availability of molecules [41]. In the colon, SOX9-high positive cells are also LGR5 [5]. However, we were not able to set up LGR5 immunostaining in rat colons to confirm this observation. In our model, R-SPONDIN (ligand of LGR5) is expressed in the colonic mucosa but its expression is not modified after MSC treatment. However, we cannot exclude the synergy of R-SPONDIN and WNT molecules in inducing non-canonical signaling and ISC proliferation [40], [41].

Although various drugs have been already been clinically tested to reduce “pelvic radiation disease”, no agents have been identified that are able to prevent or reduce intestinal radiation toxicity, which is the most important dose-limiting factor during pelvic radiotherapy [10]. This study provides evidence for the potential of MSC therapy to limit the effects of radiation on the colon through enhancement of the regenerative process. A specific feature of “pelvic radiation disease” is that other organs such as the bladder may also be altered in the long term, leading to intensified side effects [1]. In this case, MSC treatment provides a significant therapeutic advantage since its use in regenerative medicine for the restoration of many organs (bladder, liver, kidney, etc.) has been widely described [12], [13].

The therapeutic efficacy induced by MSC might require boosted proliferation of epithelial cells, including some that are in the field of irradiation. The stimulation of these irradiated cells might lead over time to malignancies. Moreover, MSC also might enhance the growth of pre-existing cancer. Studies analyzing MSC’s effects on tumorigenesis in different models had mixed and controversial results. Additional studies are underway in our laboratory to study MSC side effects in a rat colorectal adenoma model after tumor reduction by radiation. This complementary work is needed to offer new therapeutic perspectives for the treatment of patients suffering from late-onset damage induced by pelvic radiotherapy.

### Conclusions

During radiotherapy protocol, healthy tissue located near the tumor can be affected by ionizing radiation leading to severe tissue damage. In this study, we demonstrated that MSC therapy reduces irreversible radiation-induced colonic ulcers and proposed mechanisms of therapeutic efficacy. MSC engraft in lung and colonic mucosa also mobilize endogenous MSC that could have lasting benefits over time. This new insight of MSC action is of major interest and further investigations are necessary to test the mobilization of other progenitor cells. We also demonstrated that MSC therapy stimulates proliferation and growth factor secretion of colonic epithelial cells positive for SOX9 progenitor/stem cell markers. Therefore, the therapeutic benefit of stem cell therapy using MSC is induced by stimulating endogenous host progenitor cells to improve the regenerative process.
